# The usage of data in NHS primary care commissioning: a realist evaluation

**DOI:** 10.1186/s12875-023-02193-4

**Published:** 2023-12-14

**Authors:** Alexandra Jager, Chrysanthi Papoutsi, Geoff Wong

**Affiliations:** Nuffield Department of Primary Care Health Sciences, University of Oxford, Oxford, UK

**Keywords:** Realism, Commissioning, Primary care

## Abstract

**Background:**

To improve health outcomes and address mounting costs pressures, policy-makers have encouraged primary care commissioners in the British National Health Service (NHS) to increase the usage of data in decision-making. However, there exists limited research on this topic. In this study, we aimed to understand how and why primary care commissioners use data (i.e. quantitative, statistical information) to inform commissioning, and what outcomes this leads to.

**Methods:**

A realist evaluation was completed to create context-mechanism-outcome configurations (CMOs) relating to the contexts influencing the usage of data in primary care commissioning. Using a realist logic of analysis and drawing on substantive theories, we analysed qualitative content from 30 interviews and 51 meetings (51 recordings and 19 accompanying meeting minutes) to develop CMOs. Purposive sampling was used to recruit interviewees from diverse backgrounds.

**Results:**

Thirty-five CMOs were formed, resulting in an overarching realist programme theory. Thirteen CMOs were identical and 3 were truncated versions of those formed in an existing realist synthesis on the same topic. Seven entirely new CMOs, and 12 refined and enhanced CMOs vis-à-vis the synthesis were created. The findings included CMOs containing contexts which facilitated the usage of data, including the presence of a data champion and commissioners’ perceptions that external providers offered new skillsets and types of data. Other CMOs included contexts presenting barriers to using data, such as data not being presented in an interoperable way with consistent definitions, or financial pressures inhibiting commissioners’ abilities to make evidence-based decisions.

**Conclusions:**

Commissioners are enthusiastic about using data as a source of information, a tool to stimulate improvements, and a warrant for decision-making. However, they also face considerable challenges when using them. There are replicable contexts available to facilitate commissioners’ usage of data, which we used to inform policy recommendations. The findings of this study and our recommendations are pertinent in light of governments’ increasing commitment to data-driven commissioning and health policy-making.

**Supplementary Information:**

The online version contains supplementary material available at 10.1186/s12875-023-02193-4.

## Introduction

NHS primary care commissioning involves the planning, contracting, and monitoring of primary care services (GP, dentistry, pharmacy, and ophthalmology services) [1, 2]. Although the NHS has a documented ‘commissioning cycle’ consisting of nine discrete steps and activities, [3] in practice, commissioning is ‘messy and fragmented,’ [4] and only some activities undertaken by commissioners overlap with the official nine-step ‘commissioning cycle’ [5]. General practice deals with around 90% of NHS contacts while receiving under 10% of the budget, but is facing myriad pressures, including increasing patient demands [6]. Primary care services are essential to a sustainable health service, but their commissioning is complex and frequently reformed [7].

The 2012 Health and Social Care Act mandated research evidence as a core consideration in commissioning [8]. NHS England has created tools and guidance to facilitate evidence-based commissioning [9]. Evidence can be defined as any form of information that can be used for making judgements or decisions [10] and be split into quantitative evidence (henceforth ‘data’ in the context of this study) and qualitative evidence [11]. The defining hallmarks of quantitative evidence are its ability to express things in numerical form across multiple cases. Examples of quantitative evidence (i.e. data) include data from public health surveillance systems, evidence from Cochrane reviews, and surveys, whereas qualitative evidence involves nonnumerical observations including interviews, focus groups, and observations [11]. Examples of datasets NHS commissioners use include NHS RightCare and the NHS Atlas of Variation in Healthcare, which contain various types of variation data including spend and prescribing data [12, 13].

A commitment to using data in commissioning has been cemented in recent policy documents, including the ‘Next Steps on the NHS Five Year Forward View’ (2017), and many Sustainability and Transformation Plans (which aimed to implement the 2014 Five Year Forward View) [14, 15]. Recently formed Integrated Care Systems (ICSs) use connected datasets to facilitate health improvements [16]. However, the literature on this topic has criticised the quality and utility of the data available, and noted commissioners’ challenges using them [12, 14, 17]. A recurring conclusion is that more attention should be paid to contextual factors if evidence use is to be understood and increased [18–20].

This study aimed to understand how and why primary care commissioners (henceforth commissioners) use data to inform commissioning, what outcomes this leads to, and what factors facilitate and inhibit the usage of data in primary care commissioning decisions. It builds on our realist synthesis (systematic review) on the same topic [21] by comparing the findings, highlighting overlaps and new insights and nuances.

## Methods

Realist evaluations aim to understand how policies and interventions work, who they work for, and in what circumstances by analysing primary sources [22] and creating context (C) + mechanism (M) + outcome (O) configurations (CMOs) showing how an outcome is achieved through the triggering of an underlying mechanism in specific context(s) [23]. By developing and refining CMOs, researchers develop causal explanations about why programmes and interventions work (or don’t), and why the same programme resources might be acted upon in different ways by different participants in different contexts [23, 24]. In realism, contexts can be material, social, and psychological, and are the features of situations that affect the triggering of programme mechanisms [25]. Underlying causal processes known as mechanisms often cannot be seen or directly observed, even if they lead to an observable outcome, and provide explanations of why change occurs [26]. Realist evaluations culminate in a realist programme theory comprising one or more CMOs [27]. This publication follows the RAMESES reporting standards for realist evaluations [22].

### Data collection

This evaluation is based on primary data from interviews and meetings collected between November 2020 and April 2022, hence spanning much of the COVID-19 pandemic.

#### Recruitment and data collection: interviews

A realist sampling strategy should enable the testing of contexts [28] and interviewees should be selected based on their ability to confirm, falsify, and refine theory [29]. Selecting dissimilar settings enables the discovery of common and unique concepts across different contexts [30]. Purposive sampling was used: 23 interviewees were commissioners selected to ensure diversity based on the financial performance and geographic location of their respective Clinical Commissioning Group (CCG), and job titles. Seven academic experts on evidence-based commissioning were interviewed, defined as people who had published research on evidence-based commissioning and had experience of collecting and analysing primary data such as meeting observations on this topic.

Commissioners were selected for interview due to their first-hand, in-depth knowledge. Academic experts were chosen partly because recruiting NHS employees was challenging during the COVID-19 pandemic, but also because they had had the opportunity to observe commissioners in person. Commissioners were recruited using existing contacts, snowballing, and by contacting CCGs. Experts were recruited via email.

#### Data collection: meetings

During the COVID-19 pandemic, some CCGs began making the recordings of and minutes from their Primary Care Commissioning Committee meetings publicly available online. All CCG websites in England as well as YouTube and Google were searched to identify as many meeting recordings and minutes as possible between June 2020 and April 2022. The portions of the meeting recordings related to data usage in commissioning were transcribed and analysed.

### Data analysis

**Fig. 1:**
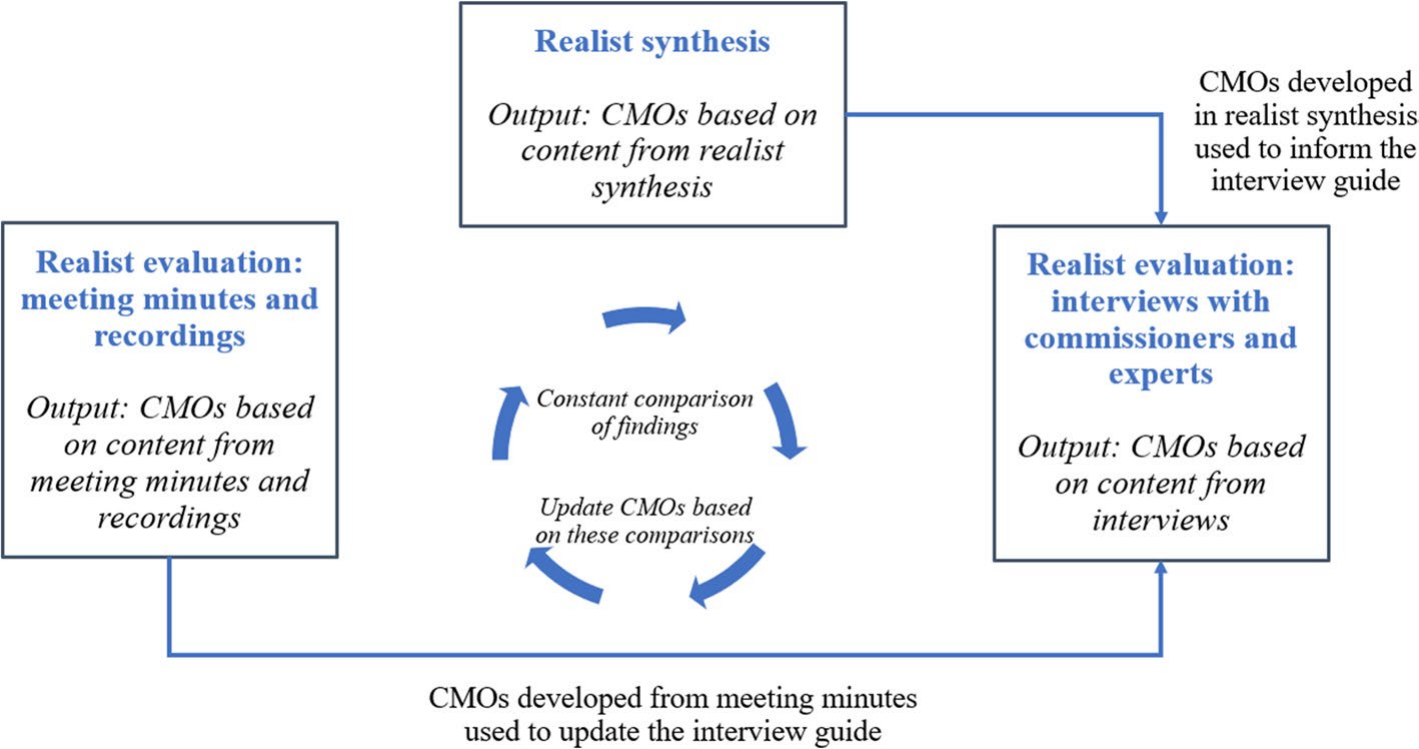
Constant comparison approach

All content was analysed according to a realist logic of analysis and linked to substantive theories where possible [27]. These substantive theories were found in the realist synthesis, the broader realist methodological literature, recommendations from other researchers and readings of the broader economic, social, and psychological literature. Realist evaluations should include an initial programme theory, i.e. a description of how a programme is thought to work and why, at the outset to facilitate the development of CMOs [31, 32]. This realist evaluation began once a synthesis on the same topic [21] was already underway. This synthesis included a draft programme theory consisting of hypotheses in a non-realist format about the contexts influencing commissioners’ usage of data (Additional file 1). Many of these hypotheses were later adapted into full CMOs and accelerated CMO formation by providing an initial framework for CMO development. In addition, the CMOs from the synthesis served as a de facto programme theory for using the constant comparison approach: analysis of the three content sources (synthesis, interviews, and meetings) was conducted using an interactive convergent design, meaning content from different sources was analysed in a similar timeframe [33] and interactively so that the developing findings could ‘talk’ to each other [34]. This was used in conjunction with the constant comparison method, which prescribes the ongoing comparing and contrasting of findings across different sources and asks the researcher to consider what information found using one source could add to another (Fig. 1) [35]. Specifically, this included the following activities:

- Using substantive theories discovered in the realist review to develop CMOs based on the meeting and interview content.
- Using findings from the realist synthesis and content from meetings to inform the interview guide, which was updated on an ongoing basis.
- If a CMO was found in one content source but not another, the researchers re-read all the content sources in which the CMO was apparently not present to ascertain if the evidence for a CMO had been missed in the initial analysis.
- Pawson’s method of reconciliation: where apparently contradictory findings were found i.e. (partially) contradictory CMOs across the content sources, these were further investigated to by re-reading and re-analysing the content sources [36, 37]. Upon re-reading and re-analysing the content, it was usually discovered that what initially looked like a contradiction could be explained and reconciled by the development of more nuanced and refined CMOs.

The CMOs developed were richer than they would have been had the CMOs been developed noninteractively, or had only one content source been used to develop CMOs, even when the same CMO was found across all sources. Furthermore, some argue congruence and/or complementarity of findings across disparate sources is desirable, since having multiple independent assessments come to the same conclusion strengthens credibility [38].

Interviews were conducted using the realist interviewing approach where the researcher’s theory is the subject matter of the interview, and the interviewee is there to confirm, falsify, and refine the theory [39]. We based the interview questions (Additional file 2) on a three-phase interview model whereby realist theories and CMOs were progressively refined and fine-tuned [28, 40]. In preparation, three interviews with lay members of health services commissioning organisations were conducted to preliminarily explore the types of evidence, and in particular data, they had used and observed being used during their involvement in commissioning. Interviewees were also asked to comment on the phrasing of interview questions and suggest improvements.

## Results

### Overview of content from primary sources

#### Interviewees

Thirty people were interviewed (23 commissioners and 7 experts) (Additional file 3).

#### Meetings

Content from 51 meetings was analysed (51 meeting recordings and 19 accompanying sets of minutes across 18 CCGs) (Additional file 4).

### Contexts, mechanisms, and outcomes

A total of 35 CMOs were created based on the content from interviews and meetings. Sixteen were identical to or truncated versions of those found in the synthesis (Additional file 6), 7 CMOs were new, and 12 CMOs had some degree of similarity to those in the synthesis but the content from interviews and meetings provided additional insights and nuances. The substantive theories used to develop the 19 new and revised CMOs are listed in additional file 5.

The CMOs have been grouped into CMOs related to facilitating contexts (contexts promoting commissioners’ usage of data, highlighted green) and inhibiting contexts (contexts triggering mechanisms that made the usage of data unlikely, highlighted orange). Whilst some of the outcomes identified were dichotomous (i.e. data were either used or not to inform decision-making), others are more nuanced as they e.g. outline the alternative forms of evidence commissioners may seek if data are unavailable or deemed unsuitable, or the ways in which commissioners use data as a tool or warrant. The mechanisms identified provide insight into the ‘why’ i.e. the reasoning contexts triggered amongst commissioners that caused them to use (or not use) data a certain way.

#### CMOs that were identical or truncated versions of those in the realist synthesis

We were able to directly validate 16 CMOs (CMOs 1–16) from the realist synthesis. Thirteen of these were CMOs developed from the meeting and/or interview content identical to those in the synthesis (Additional file 6), and three were truncated versions of CMOs from the synthesis, i.e. part of the CMO present in the synthesis was not present in the CMOs developed based on the interview/meeting content. This provides additional robustness and validity to the synthesis’s findings.

#### New CMOs based on interview and meeting content

Seven new CMOs (CMOs 16–22) vis-à-vis the synthesis were developed, i.e. these CMOs were not present in the synthesis (Table 1). Six of these were based solely on interview content, and one CMO on meeting content.

**Table 1:**
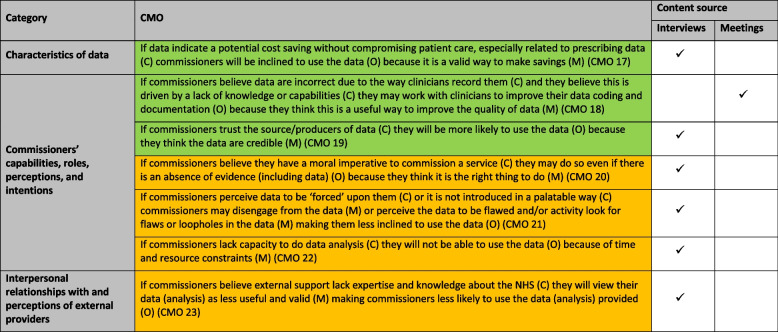
New CMOs developed

Green background = facilitating contexts, orange text = inhibiting contexts

##### New CMOs: facilitating contexts

If data indicated a potential cost saving without compromising patient care (often related to prescribing data), commissioners were inclined to use the data, believing this was a valid way to make savings (CMO 17). Commissioners sometimes had access to data they thought were incorrect due to the way clinicians coded, documented, or reported data. The errors in the data were presumed to be due to gaps in knowledge or capabilities, and commissioners believed that offering clinicians help and support was a good way to improve the quality of data, leading them to work with clinicians to achieve improvements (CMO 18).

*‘Because I think with any incident reporting it can become a bit of…a kind of perception of a lot of process and very little return. So I think the one thing I would absolutely be pushing is that we show the difference that it makes by reporting incidents and really starting to encourage more of the lessons learned and why it makes a difference and why it’s important.’*(Commissioner 21 – A, Meeting 21, Agenda item: Primary Care Quality Report).

Trusting the source of data or their owner increased commissioners’ belief that data were credible, in turn making them more likely to use the data (CMO 19). For example, one interviewee lauded Open Prescribing, which was ‘led by clinicians’ and being from a ‘good data source’ facilitated the data’s usage:

*‘It’s up to date, it’s user friendly, that’s why I think we tend to look at it and in a lot of ways because it’s driven by pharmacists who understand what are the things that we need to know…’*(Commissioner interview 4, Deputy Director of Finance for a CCG).

##### New CMOs: inhibiting contexts

If commissioners believed they had a moral imperative to commission a service, this was sometimes done even if there was an absence of evidence (including data), since it was the ‘right thing’ to do (CMO 20). If commissioners felt data had been ‘forced’ upon them or not introduced in a palatable way, they disengaged, sometimes actively looking for flaws or loopholes in the data, and were thus less inclined to use them (CMO 21):

*‘…RightCare was a methodology that was being pushed on us from, from a central perspective as well and it was used as very crudely as benchmarking…without doing the bit about bringing in clinicians with you…it drove a lot of people to be… almost dis-interpreting the data i.e. finding where the loopholes were, why it was wrong….’*(Commissioner interview 4, Deputy Director of Finance for a CCG).

A lack of capacity to do data analysis left commissioners unable to use data (CMO 22). Commissioners believing that external support lacked NHS-specific knowledge and expertise made commissioners view the data (analysis) produced by them as less valid and useful (CMO 23):

*‘So yes, somebody coming in to actually help you to work out your problems and you solve your problems works but I remembered yes, there was a lot of consultancy work at the beginning of CCGs and PCT days and it was highly ineffectual….it was actually a good way of throwing money at something and then and then finding that it did not fix the problem. The trouble with experts, they’re often not experts. They often haven’t actually worked in the field.’*(Commissioner interview 14, clinical commissioner (GP).

#### CMOs refined based on interview and meeting content

Twelve CMOs developed from the interview and meeting content had some degree of overlap or similarity to those in the synthesis, and were refined or expanded using content from meetings and interviews. The following typographical emphasis (bold, italics, underline, etc.) are used to show how these CMOs compare to those from the synthesis:

- Portions of CMOs without any typographical emphases indicate convergence of findings, i.e. the portion of the CMO identical to the synthesis.
- Bold text indicates a new finding compared to the synthesis.
- Italics indicate a complementary finding, i.e. a similar finding to the synthesis.
- Crossed-out text indicates information that was present in the synthesis but is not present in the meeting or interview CMO (note: where complementary findings are present, italics are used in lieu of crossed-out text, since the findings are sufficiently complementary/similar).

##### Refined CMOs: facilitating contexts

The eight refined CMOs related to facilitating contexts are presented in Table 2.

**Table 2:** Refined CMOs: facilitating contexts

**Category**	**CMO #**	**Interviews**	**Meetings**
Steps of the commissioning cycle	CMO 24	*If commissioners want to identify commissioning priorities (C) they may consult data as a starting point (O) since they believe they are useful (M)*	Silence (CMO not present)
	CMO 25	If commissioners believe that clinicians or service providers are not aware their performance is below average (C) and they do not want to be perceived as ‘judgemental’ or ‘performance managing’ they may share data with them in the hope of stimulating improvements (O) because of a perception that sharing data can empower clinicians or service providers to come up with solutions (M)	If commissioners believe that clinicians or service providers are not aware their performance is below average (C) they may share data with them in the hope of stimulating improvements (O) *based on the assumption sharing this data may trigger discomfort, pressure, and awareness and therefore improvements (M)*
Characteristics of data	CMO 26	If commissioners have access to combined datasets (C) they will be more likely to use them (O) because the data are useful (M) and because commissioners can gain a fuller understanding of the patient journey (M) **and because the data facilitate integrated commissioning (M)**	If commissioners have access to combined datasets (C) they will be more likely to use them (O) because the data are useful (M) and because commissioners can gain a fuller understanding of the patient journey (M) **because the data facilitate integrated commissioning (M) and because they have reassurance that the information is comprehensive (M)**
	CMO 27	Presenting key pieces of data in a succinct, **visually appealing**, and easily digestible manner to commissioners (C) can increase the likelihood the data will be used (O) since this increases their engagement with and understanding of data (M)	Silence (CMO not present)
	CMO 28	If commissioners have access to flawed or imperfect data they understand the limitations of (C) and this is the only type of data they have access to (C) they will still try to use the data (O) because they can adapt them in ways that are useful while taking into account the data’s limitations (M) and they believe this is better than using no data at all (M) **and the data make decisions more defensible (M)**	In a context where commissioners have access to ‘imperfect’ data they understand the limitations of (C) and this is the only type of data they have access to (C) **and/or they have a commissioning issue they (urgently) want to address and/or (C) they feel they have an obligation to use the data (C)** they will still try to use the data (O) because they can adapt them in ways that are useful while taking into account the data’s limitations (M) and they believe this is better than using no data at all (M)
	CMO 29	Having a data champion within the commissioning team support and promote the usage of data in commissioning decisions (C) can increase the usage of data (O) because the data champion can increase engagement and persuade people to use data (M) **and because commissioners are more receptive to communication about the importance of data if it comes from a team member (M)**	Silence (CMO not present)
Commissioners’ capabilities, roles, perceptions, and intentions	CMO 30	If commissioners want to persuade others about their commissioning proposals (C) they may (selectively) use data to support their proposals (O) due to a perception that they are a form of evidence that are ‘objective’ and can increase the legitimacy of proposals (M)	If commissioners want to persuade **or reassure** others about their commissioning proposals (C) they may (selectively) use data to support their proposals (O) due to a perception that it is a form of evidence that is ‘objective’ and can increase the legitimacy of proposals (M) **they may stress that these are based on data (O) since commissioners believe data are useful to advocate and justify their proposals (M)**
Interpersonal relationships with and perceptions of external providers	CMO 31	If commissioners perceive external support to be able to provide different or new skills, **data, or additional capacity** (C) commissioners will be more inclined to use the data and outputs they produce (O) because they are perceived as novel and useful (M) **and the commissioners believe this is a worthwhile financial investment (M)**	Silence (CMO not present)

Interviewees described using data to identify commissioning priorities, although they stressed that data could only provide a ‘starting point,’ and that commissioners would need to complete additional analysis to develop commissioning plans (CMO 24). One commissioner described using RightCare data to identify areas for further investigation:

*‘So we know what areas for example we’re an outlier in in terms of usage and in terms of cost and those things. What we found with that kind of data is, it doesn’t necessarily tell you where you could make, it tells you the areas to look at but you usually need to do a bit more digging to understand what it is that the data is telling you’.*(Commissioner interview 15, Manager)

Interviewees stated they shared data to ‘start conversations’ with clinicians and service providers who were unaware their performance was below average, thereby enabling them to develop their own solutions and draw their own conclusions (CMO 25). The meeting content was similar, except that commissioners aimed to elicit feelings of discomfort and pressure in addition to awareness by sharing data:

*‘I think sharing the data with them and even if it’s on a one-to-one basis I think that was the most powerful thing before yeah so I think I think it can sometimes the messages can sometimes get lost in all the messages that are out there so I think actually having very specific data around very specific practices whilst it’s not comfortable I think absolutely does it does get action.’*(Commissioner 8-B, Meeting 8, agenda item: antibiotic prescribing).

Combined datasets, i.e. those combining data from different sources, e.g. from primary and secondary care, were perceived as useful, enabling commissioners to gain a fuller understanding of the ‘patient journey’ (CMO 26). These data facilitated integrated commissioning, and, in the meetings content, were noted to provide assurance that information was comprehensive. Sharing data in a succinct and visually appealing manner such as via dashboards, infographics, or maps with hotspots increased engagement (CMO 27):

*‘you take a document and it is pulled together by people who really know how to take data and how to set up a document, people look at that and go, “Oooh.” They’ll make their decisions on, ‘Is it easy to read? Is it well presented? Is it a, a glossy document?’… and it’s very visually helpful and to be perfectly honest to try and get people to understand things, if you can’t put a pretty picture in front of them it just doesn’t work. Give them a table of data and numbers and they will cringe. Give them a pretty picture in a diagram and they’re all happy…’*(Commissioner interview 10, Director, CCG).

Where commissioners only had access to flawed or imperfect data they understood the limitations of, they still tried to use the data, since they could adapt them in ways that were useful while taking into account the data’s limitations, which was considered better than using no data at all (CMO 28). Interviewees added that this could make their decisions more defensible. In meetings, this was sometimes also used in a context of having commissioning issues that needed to be urgently addressed or when commissioners felt an obligation to use data. Having a data champion, especially one who was part of the commissioning team, facilitated the usage of data, since the champion could increase engagement and persuade people to use data, and commissioners were more receptive to communication about data’s importance if it came from a team member (CMO 29). Interviewees described using data as a source of evidence to persuade others and justify proposals, due to perception that data were ‘objective’ and could increase the legitimacy of proposals (CMO 30). In meetings, where commissioners wanted to persuade or reassure others about their commissioning proposals they might stress they were based on data since data were useful for advocating and justifying proposals:

*‘This is something that the team have been working through this hasn’t been done just from a management perspective so to give the committee that reassurance: this has very much been driven through looking at the data with partners that we’ve been working with around population health.’*(Commissioner 18 – A, Meeting 18, agenda item: primary care strategic priorities).

Interviewees were receptive to external support able to offer different or new skills, data, or capacity, perceived as novel and useful, as well as worthwhile financial investment (CMO 31).

##### Refined CMOs: inhibiting contexts

The four refined CMOs related to inhibiting contexts are presented in Table 3. Data not captured and presented in an interoperable way with consistent definitions were difficult to use, since commissioners had difficulty drawing conclusions, doubted the data’s credibility, and, in the interviews, had difficulty following the patient’s journey (CMO 32). If commissioners suspected that commissioning data were inaccurate or contradictory, they did not use them due to lack of trust and difficulty drawing conclusions (CMO 33). Interviewees added that they sometimes sought alternative sources of evidence, including qualitative information, and feared they might be challenged and therefore lose credibility by using inaccurate data. If interviewees perceived a mismatch between data and clinical experience, this could lead to mistrust and therefore disengagement (CMO 34). Similarly, in meetings, commissioners cast doubt on the accuracy and trustworthiness of some data based on their own clinical knowledge as well as knowledge obtained from clinicians. When subject to financial pressures, commissioners sometimes made decisions based on little or no data, even at the expense of using data related to potential (clinical) improvements, because they felt obliged to prioritise financial issues (CMO 35).

**Table 3:** Refined CMOs: inhibiting contexts

**Category**	**CMO #**	**Interviews**	**Meetings**
**Characteristics of data**	CMO 32	When data are not captured and presented in an interoperable way with consistent definitions (C) commissioners will have difficulty using them (O) because they have difficulty drawing conclusions (M) and doubt the data’s credibility (M) **and have difficulty following the patient’s journey (M)**	When data are not captured and presented in an interoperable way with consistent definitions (C) commissioners will have difficulty using them (O) because they have difficulty drawing conclusions (M) and doubt the data’s credibility (M)
	CMO 33	In a context where commissioners suspect that commissioning data are inaccurate or contradictory (C) they will not use them in commissioning decisions (O) **and may seek alternative sources of evidence, including qualitative information (O)** because they do not trust them (M) and have difficulty drawing conclusions (M) **and they fear they may be challenged and lose credibility (M)**	In a context where commissioners suspect that commissioning data are inaccurate or contradictory (C) they will not use them in commissioning decisions (O) because they do not trust them (M) and have difficulty drawing conclusions (M)
	CMO 34	In a context where (clinical) commissioners find that the data available contradict or are in tension with their experience and knowledge (C) commissioners may become skeptical of the data (O) because they are mistrustful (M)	In a context where (clinical) commissioners believe there is misalignment between data and their own clinical experience **or information received from other clinicians** (C) commissioners may become skeptical of the data (O) because they are mistrustful (M)
**Commissioners’ capabilities, roles, perceptions, and intentions**	CMO 35	In a context where commissioners are subjected to financial pressures (C) they may choose to make commissioning decisions based on little or no evidence (including data) **including at the expense of using data related to potential (clinical) improvements (O)** because they feel obliged to prioritise financial issues (M)	Silence (CMO not present)

## Discussion

### Summary

The final programme theory (showing only the new and refined CMOs) is shown in Additional file 7. Facilitating contexts that could likely be easily replicated by policy-makers include the availability of combined datasets, presenting data in a succinct and easily-digestible way, the presence of a data champion, and a perception that external providers offer new skillsets, data or capacity. In terms of specific commissioning activities, commissioners used data to identify commissioning priorities based on a belief that data were useful and shared data with clinicians to stimulate improvements with the aim of empowering them and triggering awareness, learning, and discomfort. Other facilitating contexts included commissioners using data as a warrant for convincing or reassuring others about commissioning proposals, or commissioners choosing to use flawed or imperfect data they understood the limitations of in the context of urgently needing to address a commissioning issue.

However, the final programme theory also shows that commissioners face a range of barriers to using data, including a lack of capacity, low quality, contradictory and inoperable data, as well as difficult relationships with external providers of data including perceptions that these providers lack NHS specific knowledge and experience. In addition, some commissioners felt obligated to make commissioning decisions without evidence (including data) due to financial and moral obligations.

Although there were no contradictory findings across the different content sources, certain CMOs were either only present in only one or two sources, or similar but not identical across different sources. This could be due to a range of reasons, including the Hawthorne Effect, which states that people may alter their behaviour when they know they are being observed: [41] commissioners, knowing they were being recorded, may not have wanted to make certain statements in meetings. The meeting content was the most recent (2020–2022), meaning that some CMOs present in the interviews and/or synthesis but not the meetings may no longer have been topical. All the meetings took place during the COVID-19 pandemic, so commissioners were often focussed on responding to the pandemic, potentially at the expense of more ‘typical’ commissioning activities. In addition, due to the ongoing COVID-19 pandemic, all evidence gathering was remote. Video interviews differ from in-person interviews since they can suffer from poor synchronisation, resulting in less fluid conversations, less transmission of nonverbal cues, [42] meaning interviewees might have shared more information had we completed in-person interviews.

### Strengths and limitations

The key strengths of this study are its novel contribution to the literature, the large amount of primary data gathered and analysed, as well as the level of agreement between the findings across the three data sources: although not every CMO was (fully) present in every source, no CMOs contradicted each other, and there was a reasonable degree of overlap of findings, thereby strengthening the validity and robustness of the findings.

This research was conducted remotely during the COVID-19 pandemic, meaning we could not observe the dynamics of in-person meetings. It was difficult to gauge how reflective the commissioning activities observed in meetings were typical of ‘normal’ practice. Some interviews were complicated by the challenges inherent to remote working, including slow internet connections, poor phone reception, and interruptions.

### Comparison with existing literature

The findings of non-realist research parallel this study’s findings, concluding that usage of evidence and research in policy-making is a complex, context dependent process, and that evidence is often underutilised [19, 43, 44]. Mirroring this study’s findings, realist studies have found that a range of individual, interpersonal, and environment contexts influence how research and evidence are used by policy-makers, including relationships between researchers and policy-makers [45–49]. Of these studies, two were realist evaluations on evidence use in systems similar to the NHS: a study about a knowledge translation intervention in France, [47] and a study on evidence use in a health impact assessment in Canada [48]. Although many of the CMOs in these studies bear some similarity to ours, this study focussed on one specific type of evidence (data), leading to the discovery of more detailed CMOs with nuanced outcomes. Uniquely, we have shown that evidence is not used solely as a source of information by commissioners, but also as a tool to effect change and stimulate improvements, as well as a warrant for convincing others and justifying decision-making. Overall, the commissioning of healthcare services and the related contexts is an understudied topic, [1, 50] and we believe have made a new contribution to the literature by applying the analytical lens of a realist evaluation to this topic.

Given that the literature on evidence use in commissioning is largely atheoretical, the substantive theories used to facilitate CMO development had to be sourced from the wider literature, e.g. principal-agent or reference group theory. Some of the mechanisms identified in our CMOs resonate with these broader theories, suggesting there is some predictability underpinning commissioners’ decisions.

### Implications for research and practice

In 2022, CCGs were dissolved and Integrated Care Systems, which comprise Integrated Care Boards (ICBs), were formalised as legal entities, with ICBs taking on primary care commissioning [51]. Future research could test the applicability of our CMOs to these new commissioning bodies and to a post-pandemic NHS.

The relevancy of this research has been confirmed by recent policy initiatives, including the UK government’s 2020 ‘National Data Strategy’ which stated that data could ‘revolutionise the public sector’ by creating better, cheaper, and more effectively designed services, [52] and a 2022 policy paper by the Department of Health & Social Care which pledged to use data to ‘to bring benefits to all parts of health and social care’ [53]. In 2023, the Public Administration and Constitutional Affairs Committee launched an inquiry into the collection and analysis of data by the government [54]. At an international level, the European Union’s ‘European Health Data Space’ supports the usage of health data for secondary uses including research and policy-making [55]. Some of the challenges and uncertainties surrounding these new policies parallel those identified in this study, including ensuring policy-makers have the correct skills to analyse data, dealing with a ‘deluge’ of data, ensuring secondary users of data have sufficient trust in data, and providing timely and interoperable data [52–55]. This suggests that using data to inform policy-making is rarely a straightforward process, and that the NHS could monitor developments related to data usage in policy-making both in the UK and abroad to draw on lessons learnt and best practice elsewhere.

We conclude by offering several policy recommendations based on the new and refined CMOs (Table 4):

**Table 4:** Policy recommendations

**Recommendations**	**Examples of specific activities based on interview and meeting content**	**Informed by CMOs**
Facilitate integrated commissioning	• Provide combined datasets where possible. This could include e.g. combining datasets from primary and secondary care, or combining NHS datasets with data related to inequalities such as housing or income data • Ensure data are aligned with new priorities around integrated care	CMO 26
Facilitate relationships with external providers of data	• Allow commissioners to work closely with external providers of data (analysis) to foster trust and co-production • Involve commissioners in the design, management, and, if possible, procurement of data-driven projects involving external providers	CMO 19, 23, 31
Encourage commissioners to champion and promote (new) data	• Designate a data champion internal to the commissioning committee • When introducing new data to commissioners, ask a member of the committee to introduce them where possible, and ensure commissioners’ potential reservations are taken seriously	CMO 21, 29
Facilitate commissioners’ understanding of data	• Distil key data in visually compelling ways • Ensure definitions and coding of data are as consistent as possible, and inform commissioners of any deviations	CMO 27, 32

### Supplementary Information

**Additional file 1.** Draft programme theory.

** Additional file 2.** Interview content/questions.

** Additional file 3.** Overview of interviewees.

** Additional file 4.** Meeting content analysed.

** Additional file 5.** Substantive theories used to develop new and refined CMOs only.

** Additional file 6.** CMOs validated from realist synthesis.

** Additional file 7.** Final programme theory.

## Data Availability

Requests related to the meeting content used in the study are available from the corresponding author upon reasonable request. The full interview transcripts cannot be shared to protect interviewee confidentiality.

## Abbreviations

CCG: Clinical Commissioning Group
CMOs: Context, Mechanism, Outcomes
ICBs: Integrated Care Boards
ICSs: Integrated Care Systems
NHS: National Health Service
