# Supplementary material for: The usage of data in NHS primary care commissioning: a realist evaluation
Source: BMC Prim Care. 2023 Dec 14;24:275. doi: 10.1186/s12875-023-02193-4 (PMC10720102; doi:10.1186/s12875-023-02193-4)
Supplement: Supplementary file 1 — Additional file 1. Draft programme theory. [file 12875_2023_2193_MOESM1_ESM.docx]

| **Theme** | **Summary of key points in literature/information from informal discussions** | **Potential incomplete CMO configurations** |
| --- | --- | --- |
| **Financial considerations** | - Financial constraints can temper the direct influence of research evidence on decision making^1^ - Not being able to see the financial implications of data is a barrier to using data^2,3^ - If commissioners must deliver a balanced financial plan sometimes commissioning decisions based on little or no evidence are funded^1,4^ - A core role of a commissioner is to ensure you don’t overspend, constant pressure to make financial savings (informal discussions) | - If commissioners feel pressure to make financial savings, they may make commissioning decisions not necessarily based on evidence to achieve this - If commissioners cannot see the financial implications linked to data, they may be less included to use them |
| **Political model of data usage** | - This is a political model of data usage: research is ammunition for the side that finds its conclusions supportive, and is used to ‘neutralise opponents’^5^ - Sometimes purposely sought information since they were told to take a particular course of action, e.g. national directives^4,6^ - ‘Cherry picking’ of data does happen – sometimes the idea comes first, and then data is sought to substantiate this (informal discussions) | - Commissioners may choose data selectively to support what they want to do |
| **Collaborations with external providers of data (analysis)** | - A primary care commissioner critiqued having been ‘given’ rather than ‘chosen’ a software tool to help identify individuals at higher risk of using healthcare resources e.g. hospital beds by its developers, and that they had difficulty interpreting and contextualising its outputs^7^ - Collaborations with external providers can work well or not depending on several factors including the relationships between commissioners and external providers and trust^8^ - Commissioners are not always happy with the data their receive from external providers, and sometimes feel they don’t receive the information they need^9^ | - The relationship and communication between commissioners and external providers of data (analysis) is decisive in determining whether the data will be used |
| **Knowledge of clinical vs. non-clinical commissioners** | - GPs may have different knowledge of data and information and patients than other commissioners and may challenge data based on their clinical knowledge^8,10^ | - If GP commissioners believe there is a contradiction between data and their experience, they may challenge them and be less inclined to use them |
| **Triangulation and combining of data sources** | - Triangulation of different benchmarking data was found to be helpful in identifying areas for action^3,11^ - Commissioners want to have access to combined datasets, especially at the patient level^12^ | - Combining and triangulating data sources may facilitate the usage of data |
| **Presentation of data, data overload** | - Commissioners have extensive access to evidence including national and regional directives, meeting papers, business cases, reports, patient satisfaction surveys, guidelines, pathways, and performance, activity, financial and referral data. To capture commissioners’ attention, these documents often had a summary of no more than one side of A4 with clearly bulleted action points^3,8^ | - The format in which data are presented may facilitate or hinder their usage |
| **Local vs. national data** | - Local data often trumped national or research-based information and local evaluations were seen as helpful in directly answering commissioners’ questions^4,8^ - Commissioners consider local data to be an important source of evidence^13^, sometimes this was because a local population has distinctive features^14^ | - Local data may trump national data in certain contexts |
| **Data vs. information** | - Commissioners sometimes have challenges operationalising data^3,4^ | - If commissioners cannot operationalise data, they cannot use them |
| **Variation data** | - Can be used in myriad ways to drive improvement, including by stimulating competition and peer-pressure^8,9,15^ but may also lead commissioners to offer support to practices and/clinicians^9,11,14^ | - Commissioners may use variation data in different ways depending on context |
| **Data quality** | - Some commissioners felt the data they had access to was of poor quality or untimely and that access to real-time and more timely data would be beneficial^2,3,6,8,16,17^ | - Poor quality and untimely data are unlikely to be used by commissioners |

- 1. Elliot H, Popay J. How are policy makers using evidence? Models of research utilisation and local NHS policy making. Journal of Epidemiology & Community Health 2000;54:461-8.
- 2. Curry N, Goodwin N, Naylor CD, Robertson R. Practice-based Commissioning: Reinvigorate, Replace or Abandon? 2008.
- 3. Schang L, Morton A, DaSilva P, Bevan G. From data to decisions? Exploring how healthcare payers respond to the NHS Atlas of Variation in Healthcare in England. Health Policy 2014;114:79-87.
- 4. Wye L, Brangan E, Cameron A, Gabbay J, Klein JH, Pope C. Evidence based policy making and the 'art' of commissioning - how English healthcare commissioners access and use information and academic research in 'real life' decision-making: an empirical qualitative study. BMC Health Serv Res 2015;15:430.
- 5. Weiss C. The Many Meanings of Research Utilization. Public Administration Review 1979;39:426.
- 6. Shaw SE, Smith JA, Porter A, Rosen R, Mays N. The work of commissioning: a multisite case study of healthcare commissioning in England's NHS. BMJ Open 2013;3:e003341.
- 7. Wye L, Brangan E, Cameron A, et al. What do external consultants from private and not-for-profit companies offer healthcare commissioners? A qualitative study of knowledge exchange. BMJ Open 2015;5:e006558.
- 8. Wye L, Brangan E, Cameron A, Gabbay J, Klein J, Pope C. Knowledge exchange in health-care commissioning: case studies of the use of commercial, not-for-profit and public sector agencies, 2011-14. Health Services and Delivery Research 2015;3.
- 9. Holder H, Robertson R, Ross S, Bennett LB, Gosling J, Curry N. Risk or reward? The Changing Role of CCGs in General Practice. 2015.
- 10. Miller R, Peckham S, Coleman A, McDermott I, Harrison S, Checkland K. What happens when GPs engage in commissioning? Two decades of experience in the English NHS. Journal of Health Services Research & Policy 2016;21:126-33.
- 11. Schang L, Morton A. LSE/Right Care project on NHS Commissioners’ use of the NHS Atlas of Variation in Healthcare. Case studies of local uptake. Right Care Casebook Series <http://wwwrightcarenhsuk/atlas> 2012.
- 12. Smith J, Curry N, Mays N, Dixon J. Where Next for Commissioning in the English NHS? London: Nuffield Trust; 2010.
- 13. Clarke A, Taylor-Phillips S, Swan J, et al. Evidence-based commissioning in the English NHS: who uses which sources of evidence? A survey 2010/2011. BMJ Open 2013;3.
- 14. McDermott I, Warwick-Giles L, Gore O, et al. Understanding primary care co-commissioning: Uptake, development, and impacts. Final report. PRUComm:  NIHR Policy Research Unit in Health and Social Care Systems and Commissioning; 2018.
- 15. Holder H, Robertson R, Ross S, Bennett L, Gosling J, Curry N. Risk or reward?: the changing role of CCGs in general practice. 2015.
- 16. Smith J, Shaw S, Porter A, et al. Commissioning high quality care for people with long-term conditions. Final report. Nuffield Trust; 2013.
- 17. Naylor C, Goodwin N. Building high-quality commissioning : what role can external organisations play? London: King's Fund; 2010.
