# Supplementary material for: The usage of data in NHS primary care commissioning: a realist evaluation
Source: BMC Prim Care. 2023 Dec 14;24:275. doi: 10.1186/s12875-023-02193-4 (PMC10720102; doi:10.1186/s12875-023-02193-4)
Supplement: Supplementary file 2 — Additional file 2. Interview content/questions. [file 12875_2023_2193_MOESM2_ESM.docx]

**Note: the questions listed below are examples only and not exhaustive.**

## Sample interview questions (commissioners)

Can you tell me a bit about the types of evidence, documents, or information you have used to inform your commissioning decisions?

- In your experience, how do commissioners respond to variation data? In what circumstances are variation data useful (or not) when making commissioning decisions?
  - Some CCGs appear to use prescribing data in decision making. Could you tell me a bit more about how you might use this, if at all?
- Do you have experience of working with external organisations (e.g. management consultancies or analytics providers) who provided support with analysing and/or providing data to inform commissioning decisions? When is this useful? Why?
  - How do they compare to internal BI (Business Intelligence) team? When might you use an external company vs. your internal team?
- I’ve seen examples in the literature where “data champions” - individuals who are passionate about using data effectively and who promote data best-practice - have proven themselves essential to increasing the usage of data in commissioning decisions. Is this something you’ve come across and do you think it’s effective? Why?
- Do you have any experience of gathering qualitative information (e.g. interviews or focus group outputs) to better understand data? How or when might qualitative information be helpful?
- Do you think commissioners ever perceive commissioning data to contain flaws or be of low quality? How might they react in this scenario?
- To what extent is timely access to data a challenge?
- Some CCGs are increasingly attempting to combine different datasets and use them as part of their commissioning process. Is this something you’ve come across, and why do you think this might be used?
- In some of the studies I’ve read on commissioning, commissioners have felt that financial challenges or pressures can trump data or evidence – is this something you’ve come across? What is the impact?
- In your experience, do commissioners ever have difficulty operationalising the data they have access to? Is this more common in certain contexts or certain types of data?
- In some meeting recordings I’ve listened to, commissioners appear to discuss using data to ‘nudge’ or shame certain clinicians and service providers to provide better commissioning outcomes, e.g. by presenting a primary care scorecard or prescribing data to GPs. Is this a practice you’ve come across? Do you think it is useful, and if so, what outcomes might this achieve?
- Based on the reading I’ve done it appears that commissioners are increasingly looking at data on inequalities when making commissioning decisions - to what extent does this play a role?
- To what extent do you have experience of using combined datasets in commissioning, and how or why do you find them useful?
- In your view, are there particular ways that data can be presented (or not) to commissioners that can increase engagement or understanding? Dashboards, programmes they can manipulate, etc.?
- When you reflect on the types of data that you have access to as a commissioner, are there any particular characteristics both of the data itself and how it is presented that make it particularly useful (or not) to you? Can you provide examples?
- Are there any particular challenges or scenarios relating to utilising data to inform primary care commissioning decisions that have not been covered in this conversion?

## Sample interview questions (experts)

- Do you have any observations of commissioners working with external organisations who provided them with data or data analysis? When do you think this worked well, and when did it not?
- I’ve seen examples in the literature where ‘data champions’ - individuals who are passionate about using data effectively and who promote data best-practice - have proven themselves essential to increasing the usage of data in commissioning decisions. Is this something you’ve come across?
- Some CCGs are increasingly attempting to combine different datasets and use them as part of their commissioning process. is this something you’ve come across, and why do you think this might be used?
  - [Prompt] One theory is that commissioners find this useful because it gives them a better idea of the full patient journey.
- A commonly used piece of data in commissioning are variation data. Are there specific types of variation data that you think were commonly used in commissioning, and any that you think commissioners were more sceptical of?
- What are your observations on the usage of qualitative information in commissioning (these can be ‘official’ qualitative information e.g. focus groups or interviews but also unofficial e.g. patient stories) and how this relates to data?
  - Can you think of examples where it has been used to better understand data?
- In your observations, to what extent are low quality data a challenge for commissioners?
- To what extent is access to timely data a challenge for commissioners?
- In some of the studies I’ve read on commissioning, commissioners have felt that financial challenges or pressures can trump data or evidence – is this something you’ve come across?
- Based on the reading I’ve done it appears that commissioners are increasingly looking at data on inequalities when making commissioning decisions. To what extent does this play a role?
- In your view, are there particular ways that data can be presented (or not) to commissioners that can increase engagement or understanding? Dashboards, programmes they can manipulate, etc.?
- To what extent is ‘data overload’ a challenge in commissioning?
- To what extent might commissioners’ technical skills and knowledge limit the usage of data?
