# Supplementary material for: The usage of data in NHS primary care commissioning: a realist evaluation
Source: BMC Prim Care. 2023 Dec 14;24:275. doi: 10.1186/s12875-023-02193-4 (PMC10720102; doi:10.1186/s12875-023-02193-4)
Supplement: Supplementary file 3 — Additional file 3. Overview of interviewees. [file 12875_2023_2193_MOESM3_ESM.docx]

| **Interviewee** | **Date** | **Area of commissioning/background** | **Interview duration (HH:MM:SS)** |
| --- | --- | --- | --- |
| Interviewee 1 | 06/11/2020 | Population Health and Finance Manager | 00:54:36 |
| Interviewee 2 | 09/11/2020 | Clinician, has held multiple commissioning roles including non-Executive in a PCT | 01:02:17 |
| Interviewee 3 | 12/11/2020 | GP, has held multiple commissioning roles including medicines commissioning | 01:15:53 |
| Interviewee 4 | 30/11/2020 | Deputy Director of Finance for a CCG | 00:59:09 |
| Interviewee 5 | 01/12/2020 | Multiple roles, included chair of a primary care commissioning group and Chief Executive of a Health Authority | 00:45:35 |
| Interviewee 6 | 03/12/2020 | Quality improvement work (GP by background) | 00:55:07 |
| Interviewee 7 | 17/12/2020 | 30+ years of work experience in the NHS in policy, commissioning and change management roles | 00:49:52 |
| Interviewee 8 | 23/12/2020 | Former Director of Commissioning for a CCG | 00:43:49 |
| Interviewee 9 | 28/01/2021 | Consultant for the NHS/CSUs | 00:41:44 |
| Interviewee 10 | 29/01/2021 | Director | 00:44:38 |
| Interviewee 11 | 11/03/2021 | N/a – asked for this information to be removed | 00:52:27 |
| Interviewee 12 | 30/03/2021 | Associate Director | 00:21:54 |
| Interviewee 13 | 23/08/2021 | Chair, clinical commissioner (GP) | 00:48:44 |
| Interviewee 14 | 01/09/2021 | Clinical commissioner (GP) | 00:34:29 |
| Interviewee 15 | 30/11/2021 | Manager | 00:33:22 |
| Interviewee 16 | 14/12/2021 | Assistant Director for Primary Care | 00:39:50 |
| Interviewee 17 | 25/01/2022 | Manager | 00:32:53 |
| Interviewee 18 | 21/01/2022 | Estates (Primary Care), Deputy Director | 00:33:59 |
| Interviewee 19 | 12/01/2022 | Associate Director | 00:26:59 |
| Interviewee 20 | 09/02/2022 | Head of Primary Care Commissioning, clinical background | 00:23:23 |
| Interviewee 21 | 15/02/2022 | Leadership/management role | 00:10:38 |
| Interviewee 22 & 23 (joint interview) | 09/03/2022 | Management/Director level roles (both interviewees) | 00:24:25 |

| **Interviewee** | **Date** | **Background** | **Interview duration (HH:MM:SS)** |
| --- | --- | --- | --- |
| Interviewee 1 | 16/02/2022 | Academic at a university | 00:56:58 |
| Interviewee 2 | 21/03/2022 | Former academic | 00:27:25 |
| Interviewee 3 | 30/03/2023 | Academic at a university | 00:23:09 |
| Interviewee 4 | 31/03/2022 | Academic at a university | 00:30:13 |
| Interviewee 5 | 20/04/2022 | Academic at a university | 00:20:30 |
| Interviewee 6 | 26/04/2022 | Academic at a university | 00:24:33 |
| Interviewee 7 | 28/04/2022 | Academic at a university | 00:40:38 |
