# Supplementary material for: The usage of data in NHS primary care commissioning: a realist evaluation
Source: BMC Prim Care. 2023 Dec 14;24:275. doi: 10.1186/s12875-023-02193-4 (PMC10720102; doi:10.1186/s12875-023-02193-4)
Supplement: Supplementary file 4 — Additional file 4. Meeting content analysed. [file 12875_2023_2193_MOESM4_ESM.docx]

| **Name of meeting** | **Meeting recording accessed and used in analysis?** | **Meeting minutes accessed and used in analysis?** | **Meeting abbreviation** | **Approximate duration of recording transcribed (MM:SS) (only the meeting portion related to data use in commissioning was transcribed)** | **Words transcribed for analysis** | **CCG pseudonym** |
| --- | --- | --- | --- | --- | --- | --- |
| Meeting 1 |  |  | M1 | 03:23 | 468 | CCG 4 |
| Meeting 2 |  |  | M2 | 02:39 | 513 | CCG 12 |
| Meeting 3 |  |  | M3 | 03:21 | 605 | CCG 10 |
| Meeting 4 |  |  | M4 | 06:53 | 1031 | CCG 10 |
| Meeting 5 |  |  | M5 | 17:00 | 3099 | CCG 18 |
| Meeting 6 |  |  | M6 | 05:00 | 868 | CCG 2 |
| Meeting 7 |  |  | M7 | Recording no longer available | 193 | CCG 12 |
| Meeting 8 |  |  | M8 | 01:34 | 246 | CCG 12 |
| Meeting 9 |  |  | M9 | Recording no longer available | 354 | CCG 15 |
| Meeting 10 |  |  | M10 | 03:17 | 489 | CCG 12 |
| Meeting 11 |  |  | M11 | 03:19 | 555 | CCG 10 |
| Meeting 12 |  |  | M12 | Recording no longer available | 1329 | CCG 11 |
| Meeting 13 |  |  | M13 | 00:57 | 198 | CCG 12 |
| Meeting 14 |  |  | M14 | 01:56 | 247 | CCG 8 |
| Meeting 15 |  |  | M15 | 05:14 | 711 | CCG 12 |
| Meeting 16 |  |  | M16 | 09:19 | 1554 | CCG 12 |
| Meeting 17 |  |  | M17 | Recording no longer available | 255 | CCG 1 |
| Meeting 18 |  |  | M18 | Recording no longer available | 473 | CCG 15 |
| Meeting 19 |  |  | M19 | 10:04 | 1321 | CCG 16 |
| Meeting 20 |  |  | M20 | Recording no longer available | 448 | CCG 17 |
| Meeting 21 |  |  | M21 | 02:30 | 444 | CCG 6 |
| Meeting 22 |  |  | M22 | Recording no longer available | 959 | CCG 7 |
| Meeting 23 |  |  | M23 | 02:10 | 324 | CCG 8 |
| Meeting 24 |  |  | M24 | 00:32 | 115 | CCG 12 |
| Meeting 25 |  |  | M25 | 03:41 | 676 | CCG 18 |
| Meeting 26 |  |  | M26 | 09:58 | 1263 | CCG 10 |
| Meeting 27 |  |  | M27 | 03:07 | 511 | CCG 13 |
| Meeting 28 |  |  | M28 | 02:06 | 361 | CCG 6 |
| Meeting 29 |  |  | M29 | 00:42 | 131 | CCG 9 |
| Meeting 30 |  |  | M30 | 00:34 | 127 | CCG 14 |
| Meeting 31 |  |  | M31 | Recording no longer available | 1345 | CCG 17 |
| Meeting 32 |  |  | M32 | 04:21 | 723 | CCG 18 |
| Meeting 33 |  |  | M33 | 02:04 | 379 | CCG 15 |
| Meeting 34 |  |  | M34 | 01:00 | 179 | CCG 3 |
| Meeting 35 |  |  | M35 | 07:29 | 1365 | CCG 8 |
| Meeting 36 |  |  | M36 | 00:50 | 178 | CCG 12 |
| Meeting 37 |  |  | M37 | 10:23 | 1593 | CCG 12 |
| Meeting 38 |  |  | M38 | Recording no longer available | 443 | CCG 6 |
| Meeting 39 |  |  | M39 | 03:43 | 649 | CCG 12 |
| Meeting 40 |  |  | M40 | 4:44 | 932 | CCG 5 |
| Meeting 41 |  |  | M41 | 02:11 | 291 | CCG 12 |
| Meeting 42 |  |  | M42 | 03:11 | 496 | CCG 12 |
| Meeting 43 |  |  | M43 | 11:26 | 1639 | CCG 10 |
| Meeting 44 |  |  | M44 | 3:51 | 581 | CCG 14 |
| Meeting 45 |  |  | M45 | 00:58 | 172 | CCG 5 |
| Meeting 46 |  |  | M46 | 02:17 | 448 | CCG 5 |
| Meeting 47 |  |  | M47 | 02:08 | 382 | CCG 6 |
| Meeting 48 |  |  | M48 | 10:09 | 1510 | CCG 10 |
| Meeting 49 |  |  | M49 | 0:27 | 75 | CCG 6 |
| Meeting 50 |  |  | M50 | 03:53 | 615 | CCG 12 |
| Meeting 51 |  |  | M51 | 00:49 | 135 | CCG 15 |
