# Supplementary material for: The usage of data in NHS primary care commissioning: a realist evaluation
Source: BMC Prim Care. 2023 Dec 14;24:275. doi: 10.1186/s12875-023-02193-4 (PMC10720102; doi:10.1186/s12875-023-02193-4)
Supplement: Supplementary file 5 — Additional file 5. Substantive theories used to develop new and refined CMOs only. [file 12875_2023_2193_MOESM5_ESM.docx]

| **CMO** | **Name of substantive theory** | **Notes** |
| --- | --- | --- |
| CMO 24 | Counting or quantification (Deborah Stone) | This CMO was informed by the concepts related to ‘counting’ or quantification or policy issues as outlined by Deborah Stone in her work ‘The Policy Paradox.’ |
| CMO 29 | Data champion | A data champion is a concept used to develop the CMO |
| CMO 30 | Political model of research utilisation (Weiss) |  |
| CMO 32 | Theory of interoperability |  |
