# Supplementary material for: The usage of data in NHS primary care commissioning: a realist evaluation
Source: BMC Prim Care. 2023 Dec 14;24:275. doi: 10.1186/s12875-023-02193-4 (PMC10720102; doi:10.1186/s12875-023-02193-4)
Supplement: Supplementary file 6 — Additional file 6. CMOs validated from realist synthesis. [file 12875_2023_2193_MOESM6_ESM.docx]

**List of 16 CMOs validated by the interview and/or meeting content**

Facilitating contexts (contexts promoting commissioners’ usage of data) are highlighted green and inhibiting contexts (contexts triggering mechanisms that made the usage of data unlikely) are highlighted in orange.

The following 13 CMOs formed on interview and/or meeting content were identical those found in the synthesis:

| Category | CMO # | CMO | Content source | | |
| --- | --- | --- | --- | --- | --- |
|  |  |  | **Synthesis** | **Interviews** | **Meetings** |
| **Steps of the commissioning cycle** | CMO 1 | When commissioners believe that clinicians will act to improve when comparative data are shared and scrutinised by peers (C) they will give them data so they can review and compare their performance relative to their peers (O) because they believe improvements will occur when; there is peer pressure and orientation to a reference group (M); competition (M) and; clinicians are more receptive to feedback from their peers (M) |  |  |  |
|  | CMO 2 | Where data indicate potential for improvement, and commissioners suspect that clinicians need help to achieve improvements (C) and commissioners want to be seen as supportive rather than judging (C) commissioners may offer support to outliers and underperformers (O) because they want to maintain good relationships by being perceived as supportive (M) |  |  |  |
| **Characteristics of data** | CMO 3 | When commissioners have access to data on health inequalities (C) they may perceive them as useful to achieving a policy and/or moral objective (M) making commissioners more likely to use the data (O) |  |  |  |
|  | CMO 4 | In a context where commissioners have access to data linked to cost implications (C) they will be more inclined to use them (O) and may prioritise the data over other forms of evidence (O) because the evidence aligns with what they want to understand (M) and because it aligns with a priority they consider important (M) |  |  |  |
|  | CMO 5 | When commissioners have access to data they can segment and ‘drill down’ in (C) they are more inclined to use the data (O) because they are able to create targeted and tailored commissioning decisions (M) |  |  |  |
|  | CMO 6 | In a context where commissioners feel that factors outside of clinicians’ or service providers’ control are impacting benchmarking or variation data (C) or the data do not allow for a ‘like for like’ comparison (C) they will be less included to use the data (O) because they think they are not valid (M) |  |  |  |
|  | CMO 7 | In a context where commissioners have access to more data than they can manage (C) they may feel frustrated (M) and uncertain about what to prioritise (M) meaning they are less likely to use the data (O) |  |  |  |
| **Commissioners’ capabilities, roles, perceptions, and intentions** | CMO 8 | In a context where commissioners can choose and select data and metrics they use (C) they will become more engaged and inclined to use the data (O) because they are meaningful and valid to them (M) |  |  |  |
|  | CMO 9 | Commissioners will be less inclined to utilise data (O) if they cannot operationalise them (M). This can occur in a context of not being able to transform data into ‘information’ (C) |  |  |  |
|  | CMO 10 | When commissioners lack the skills to analyse and interpret data (C) they cannot understand and draw insights from data for commissioning decisions (O) because of the knowledge gap (M) |  |  |  |
| **Interpersonal relationships with and perceptions of external providers** | CMO 11 | When external providers understand the data needs of commissioners (C) and they can ‘co-produce’ the data (analysis) (C) commissioners are more likely to use their outputs (O), because they are relevant and useful (M) |  |  |  |
|  | CMO 12 | When commissioners can develop satisfactory relationships with the external providers of data (analysis) (C) commissioners are more likely to use data resulting from these relationships and collaborations (O) because the data produced are credible (M) |  |  |  |
|  | CMO 13 | In a context where there is a real or perceived divergence of interest (C) or information asymmetry between commissioners and external providers of data (analysis) (C) this may trigger feelings of mistrust among commissioners (M) which may make them less likely to use the data (O) |  |  |  |

The following three CMOs developed based on interview and/or meeting content are shortened or truncated versions of those in the synthesis. The crossed-out text indicated the portion of the CMO present in the synthesis but not the interview or meeting content.

**CMO 14 (findings from interview and meeting content identical):**

In contexts where commissioners wish to better understand data (C) they may supplement data with qualitative information (O) ~~because of the perceived increase in the validity of data (M) and~~ due to a perception that this will give them a fuller and more meaningful understanding of the data (M)

**CMO 15:**

Interviews:

Where commissioners have access to data that show trends and developments over time (C) they will be more inclined to use them (O) because they consider this useful (M) and think this will provide a less ‘distorted’ view (M)

Meetings

Where commissioners have access to data that show trends and developments over time (C) they will be more inclined to use them (O) because they consider this useful (M) ~~and think this will provide a less ‘distorted’ view (M)~~

**CMO 16:**

Interviews

When commissioners have data that are considered ‘real-time’ or recent (C) they will be more inclined to use them in commissioning decisions (O) ~~because they find them useful for providing immediate support or enabling speedy decisions (M)~~ because they have trust that the data reflect the current situation (M)

Meetings

When commissioners have data that are considered ‘real-time’ or recent (C) they will be more inclined to use them in commissioning decisions (O) because they find them useful for providing immediate support or enabling speedy decisions (M) and they have trust that the data reflect the current situation (M)
