# Supplementary material for: The usage of data in NHS primary care commissioning: a realist evaluation
Source: BMC Prim Care. 2023 Dec 14;24:275. doi: 10.1186/s12875-023-02193-4 (PMC10720102; doi:10.1186/s12875-023-02193-4)
Supplement: Supplementary file 7 — Additional file 7. Final programme theory. [file 12875_2023_2193_MOESM7_ESM.pptx]

## Slide 1
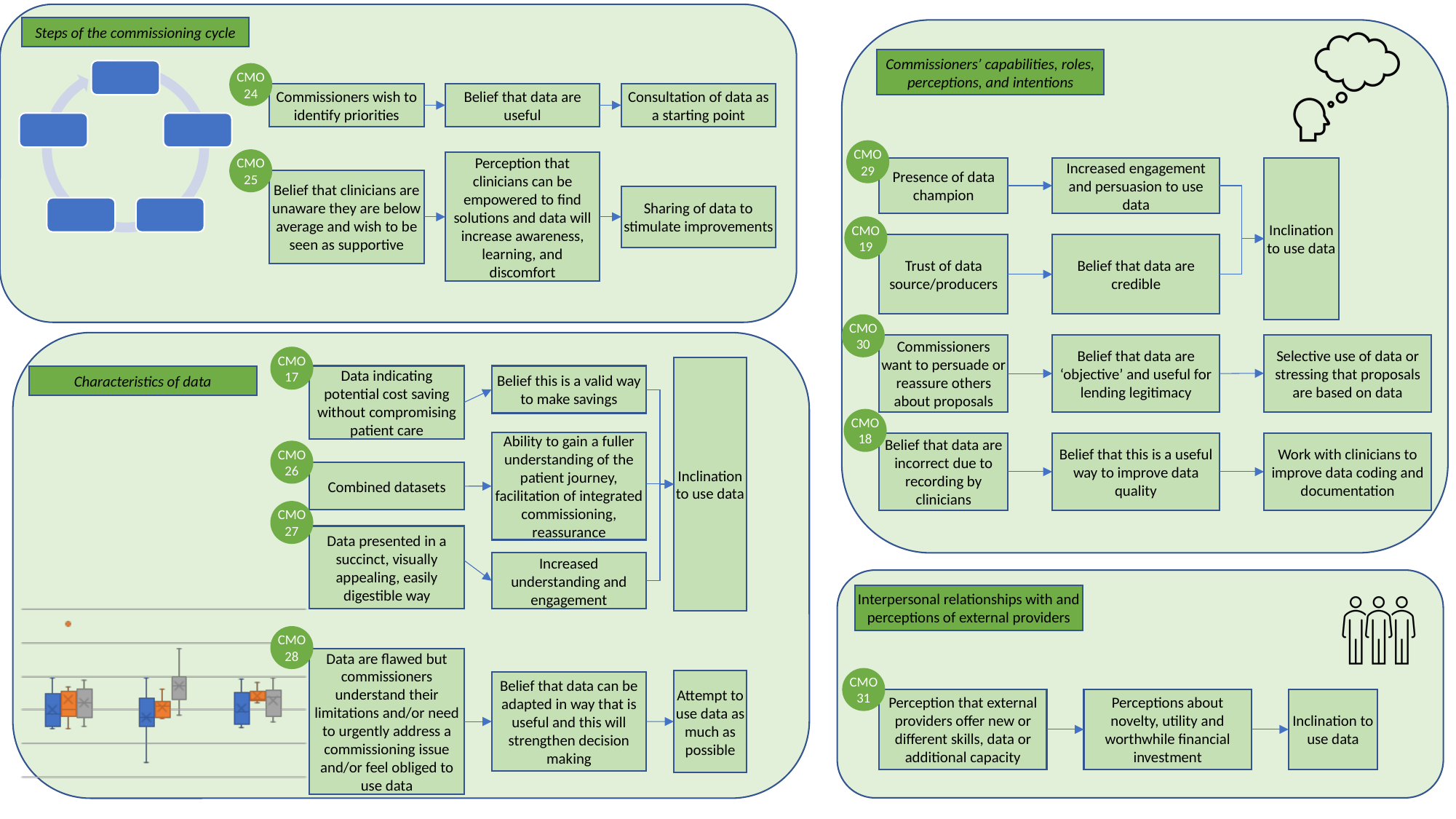

Steps of the commissioning cycle
Commissioners’ capabilities, roles, perceptions, and intentions
CMO 24
Commissioners wish to identify priorities
Belief that data are useful
Consultation of data as a starting point
CMO 29
CMO 25
Perception that clinicians can be empowered to find solutions and data will increase awareness, learning, and discomfort
Presence of data champion
Increased engagement and persuasion to use data
Inclination to use data
Belief that clinicians are unaware they are below average and wish to be seen as supportive
Sharing of data to stimulate improvements
CMO 19
Trust of data source/producers
Belief that data are credible
CMO 30
Selective use of data or stressing that proposals are based on data
Commissioners want to persuade or reassure others about proposals
Belief that data are ‘objective’ and useful for lending legitimacy
CMO 17
Inclination to use data
Data indicating potential cost saving without compromising patient care
Belief this is a valid way to make savings
Characteristics of data
CMO 18
Ability to gain a fuller understanding of the patient journey, facilitation of integrated commissioning, reassurance
Belief that data are incorrect due to recording by clinicians
Belief that this is a useful way to improve data quality
Work with clinicians to improve data coding and documentation
CMO 26
Combined datasets
CMO 27
Data presented in a succinct, visually appealing, easily digestible way
Increased understanding and engagement
Interpersonal relationships with and perceptions of external providers
CMO 28
Data are flawed but commissioners understand their limitations and/or need to urgently address a commissioning issue and/or feel obliged to use data
CMO 31
Attempt to use data as much as possible
Belief that data can be adapted in way that is useful and this will strengthen decision making
Perception that external providers offer new or different skills, data or additional capacity
Perceptions about novelty, utility and worthwhile financial investment
Inclination to use data

## Slide 2
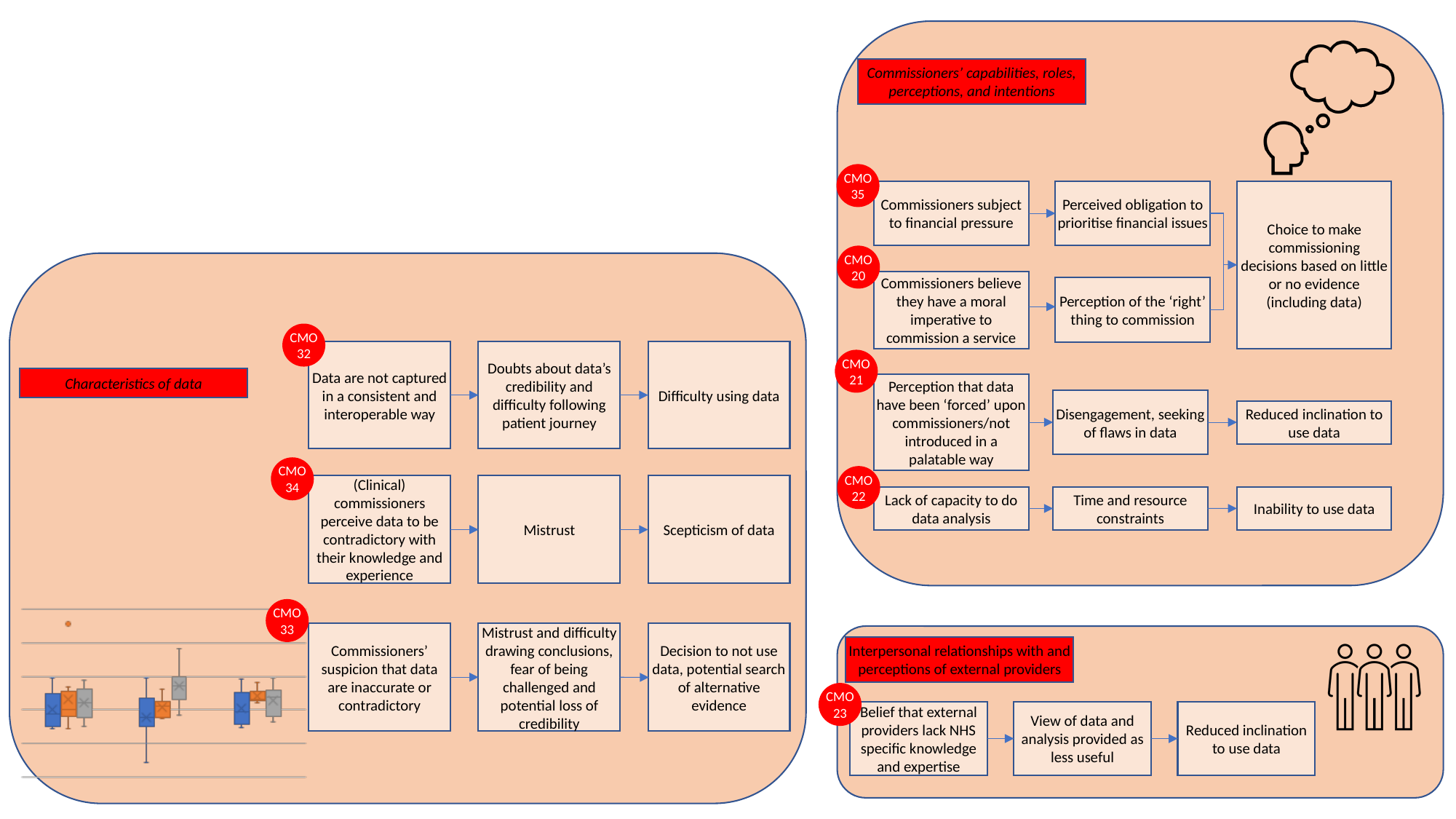

Commissioners’ capabilities, roles, perceptions, and intentions
CMO 35
Commissioners subject to financial pressure
Perceived obligation to prioritise financial issues
Choice to make commissioning decisions based on little or no evidence (including data)
CMO 20
Commissioners believe they have a moral imperative to commission a service
Perception of the ‘right’ thing to commission
CMO 32
Data are not captured in a consistent and interoperable way
Doubts about data’s credibility and difficulty following patient journey
Difficulty using data
CMO 21
Characteristics of data
Perception that data have been ‘forced’ upon commissioners/not introduced in a palatable way
Disengagement, seeking of flaws in data
Reduced inclination to use data
CMO 34
CMO 22
(Clinical) commissioners perceive data to be contradictory with their knowledge and experience
Mistrust
Scepticism of data
Inability to use data
Time and resource constraints
Lack of capacity to do data analysis
CMO 33
Commissioners’ suspicion that data are inaccurate or contradictory
Mistrust and difficulty drawing conclusions, fear of being challenged and potential loss of credibility
Decision to not use data, potential search of alternative evidence
Interpersonal relationships with and perceptions of external providers
CMO 23
Belief that external providers lack NHS specific knowledge and expertise
View of data and analysis provided as less useful
Reduced inclination to use data
